# Supplementary material for: Cultural Competence of Obstetricians/Gynecologists and Midwives Providing Midwifery Care to Roma Women in Western Greece
Source: Healthcare (Basel). 2025 Jan 19;13(2):190. doi: 10.3390/healthcare13020190 (PMC11764572; doi:10.3390/healthcare13020190)
Supplement: Supplementary file 1 [file healthcare-13-00190-s001.zip › healthcare-3377415-supplementary.pdf]

Supplementary Table S1. Sociodemographic Characteristics of Participants, Employment Details and Details Regarding the Receipt of Specialized Training for the Care of Roma Women

Parameter		N	%
**Gender**	Female	87	87.0
	Male	13	13.0
**Age, Mean value (SD)**		45 (9.7)	
**Ethnicity**	Greece	97	99.0
	Other	1	1.0
**Profession**	Midwife	81	81.0
	Doctor	19	19.0
**Educational Level**	Secondary education	6	6.0
	Bachelor's degree	63	63.0
	MSc	26	26.0
	PhD	5	5.0
**Country of Education**	Greece	96	96.0
	Abroad	4	4.0
**Workplace**	Patras	53	55.2
	Agrinio	19	19.8
	Messolonghi	12	12.5
	Preveza	12	12.5
**Professional role**	Clinical practice	89	95.7
	Administration	1	1.1
	Research	3	3.3
	Other	0	0.0
**Service of employment**	Hospital Emergency Department	3	3.0
	Maternity/Gynecology Department of the hospital	96	96.0
	Other	1	1.0
**Have you received specialized training related to the care of Roma women?**	No	90	90.9
	Yes	7	7.1
	Prefer not to answer	2	2.0
**If YES, what did the training include?**	Information on the special needs of Roma (women)	0	0.0
	Awareness of the rights of Roma (women)	1	14.3
	Information on the stereotypes concerning Roma (women)	0	0.0
	All of the above	6	85.7
**If NO, would you like to participate in such training?**	Yes, definitely	43	47.8
	Yes, under certain conditions	17	18.9

	No, not at all	9	10
	Don't know/Prefer not to answer	21	23
**If NO, what topics would you like the training to cover?**	The specific needs of Roma (women)	65	83.3
	The rights of Roma (women)	53	67.9
	Other	5	6.4

Msc: Master's degree; PhD: Doctorate; SD: standard deviation

The question assessing participants' ability to provide adequate counseling/care to Roma women had a mean score of 6.9 points (SD = 2.2 points), indicating that they evaluated their ability as moderate to high. Specifically, the mean score for health promotion among the Roma was 3.7 points (SD = 1.1 points), for referral for social care it was 3.6 points (SD = 1.2 points), and for addressing cultural differences it was 3.3 points (SD = 1.1 points). Lower average scores, indicating lesser ability, were observed in the discussion of gender-based violence, with a mean score of 3.2 points (SD = 1.2 points), and the lowest mean score of 2.4 points (SD = 1.3 points) was for the ability to discuss legal issues related to the Roma. Regarding the characteristics of the participants, there was greater agreement with the view that culture shapes human behavior and thinking, for which the mean score was 4.1 points (SD = 0.9 points).

Next in importance was the ability to adapt flexibly to a new situation, with a mean score of 3.9 points (SD = 0.9 points), followed by the recognition that one's views have a cultural background, with a mean score of 3.8 points (SD = 0.9 points), and empathy, which also had a mean score of 3.8 points (SD = 1 point). There was slightly lower agreement on the ability to understand how people feel when they come from a different culture, with a mean score of 3.7 points (SD = 1 point), and the recognition of the social needs of the Roma, with a mean score of 3.6 points (SD = 1 point). The ability to question one's own views, as well as the discomfort in doing so, both had a mean score of 3.1 points (SD = 1.2 points). Finally, stereotypes about the Roma influenced thoughts and behavior with mean scores of 3 and 2.7 points, respectively (SD = 1.2 points for both).

Supplementary Table S2. Personal Evaluation of Abilities to Provide Adequate Counseling/Care to Roma Women

Item	Mean (SD)	Median (IQR)
**To what extent do you feel capable of providing adequate counseling/care to Roma women within the scope of your duties?**	6.9 (2.2)	7 (5—9)
**To what extent do you feel capable of providing**		

**appropriate counseling/care to Roma women?**		
Health promotion among the Roma	3.7 (1.1)	4 (3 — 4)
Referral for social care	3.6 (1.2)	4 (3 — 5)
Addressing cultural differences	3.3 (1.1)	3 (3 — 4)
Discussion on gender-based violence	3.2 (1.2)	3 (3 — 4)
Legal issues related to the Roma	2.4 (1.3)	2 (1 — 3)
**For each of the following characteristics, to what extent do you believe they apply to you?**		
Culture shapes human behavior and thinking	4.1 (0.9)	4 (4 — 5)
I adapt flexibly to a new situation.	3.9 (0.9)	4 (3 — 5)
I recognize that my views have a cultural background.	3.8 (0.9)	4 (3 — 4)
I can easily empathize with another person's situation.	3.8 (1)	4 (3 — 5)
I can easily understand how people feel when they come from a different cultural/religious background	3.7 (1)	4 (3 — 4.5)
I recognize the social needs of the Roma.	3.6 (1)	4 (3 — 4)
I am able to question my own views.	3.1 (1.2)	3 (2 — 4)
I don't like questioning my own views.	3.1 (1.2)	3 (2 — 4)

My thoughts are influenced by stereotypes about the Roma.	3 (1.2)	3 (2 — 4)
My behavior is influenced by stereotypes about the Roma.	2.7 (1.2)	3 (2 — 4)

SD: standard deviation; IQR: interquartile range

The statement agreement, with a mean score of 4.5 points (SD = 0.7 points), was regarding the statement that participants could easily recognize if someone is Roma. This was followed by a mean score of 4.4 points (SD = 0.8 points) for the view that, as professionals, they would definitely provide care/support to a Roma woman, and the opinion that Roma have the same rights to access healthcare services as other citizens, with an a mean score of 4.2 points (SD = 1.1 points). There was also high agreement with the view that most health issues of Roma women are due to their cultural specificities, with a mean score of 3.8 points (SD = 1.1 points), and the opinion that prejudices against Roma are a serious social problem, with a mean score of 3.8 points (SD = 1.2 points). The recognition of Roma rights had a mean agreement score of 3.7 points (SD = 1 point), while the opinion that Roma should be hospitalized in different wards had a mean agreement score of 3.2 points (SD = 1.5 points). There was less agreement with the statement that most colleagues do not know how to provide care/support to Roma, with a mean score of 2.4 points (SD = 1.4 points). Finally, the lowest agreement was observed with the views that Roma women should receive support services and be examined by Roma professionals, with mean agreement scores of 1.9 and 1.8 points, respectively (SD = 1.2 points and SD = 1 point, respectively).

Supplementary Table S3. Responses Regarding the Provision of Healthcare Services to Roma

Item	Mean (SD)	Median (IQR)
In my profession, I can easily tell if someone is Roma.	4.5 (0.7)	5 (4 — 5)
As a professional, I would definitely provide care/support to a Roma woman.	4.4 (0.8)	5 (4 — 5)
Roma have the same rights to access healthcare services as other citizens.	4.2 (1.1)	5 (4 — 5)
Most health issues of Roma women are due to their cultural specificities.	3.8 (1.1)	4 (3 — 5)

Prejudices against Roma are a serious social problem.	3.8 (1.2)	4 (3 – 5)
I recognize the social needs of the Roma.	3.7 (1)	4 (3 – 4)
Roma should be hospitalized in different wards.	3.2 (1.5)	3 (2 – 5)
In my profession, most colleagues do not know how to provide care/support to Roma.	2.4 (1.4)	2 (1 – 4)
Roma women should receive support services from Roma professionals.	1.9 (1.2)	1 (1 – 3)
Roma women should be examined by Roma healthcare professionals.	1.8 (1)	1 (1 – 2)

SD: standard deviation; IQR: interquartile range

Supplementary Table S4. Frequency of Communication Problems with Roma During the Provision of Care

Parameter	N	%
**In the last 6 months, how many Roma women have you provided counseling/care for with whom there was a communication problem?**		
None	14	14.0
1-5	33	33.0
5-10	22	22.0
>10	31	31.0

**When providing services to Roma women, how often do you encounter communication problems?**		
Always	14	14.0
Often	53	53.0
Sometimes	26	26.0
Rarely	6	6.0
Never	1	1.0
**Feeling**	**Mean (SD)**	**Median (IQR)**
Understanding	3.6 (1)	4 (3 — 4)
Empathy	3.6 (0.9)	4 (3 — 4)
Interest	3.6 (1)	4 (3 — 4)
Respect	3.2 (1.1)	3 (3 — 4)
Other	2.6 (1.7)	2 (1 — 4)
Pity	2.1 (1.1)	2 (1 — 3)
Aversion	2 (1.1)	2 (1 — 3)
Embarrassment	2 (1.1)	2 (1 — 3)
Indifference	1.7 (0.9)	1 (1 — 2)

SD: standard deviation; IQR: interquartile range

Supplementary Table S5. Issues for Which Roma Women Visit Participants

**Issue**	**N**	**%**
Perinatal care (pregnancy monitoring, childbirth, and postpartum care)	70	70.7
Pregnancy complications	62	62.6
Postpartum complications	45	45.5

Concerns about unwanted pregnancy	35	35.4
Sterilization	35	35.4
Information about pregnancy-related issues	30	30.3
Information about access to financial support/benefits	26	26.3
Injuries	24	24.2
Problems/concerns with newborn care	15	15.2
Problems with breastfeeding	15	15.2
Domestic violence/rape	15	15.2
Prevention (e.g., Pap test, mammography)	15	15.2
Childhood vaccinations	14	14.1
Health issues (respiratory, cardiovascular, dermatological, musculoskeletal, chronic disease care)	14	14.1
Contraceptive options	11	11.1
Concerns about hereditary diseases	10	10.1
Information about housing issues	10	10.1
Information about health improvement (e.g., smoking cessation, nutrition)	8	8.1
Vaccination	6	6.1
Psychological problems (e.g., depression/anxiety)	4	4.0

Supplementary Table S6. Problems Faced by Healthcare Professionals While Providing Care to Roma Women

Problem/Access	N	%
**During the provision of support/counseling/care to Roma women, did you encounter any of the following problems?**		
They did not understand the information I was trying to convey.	70	72.9
I couldn't help them because they didn't have the necessary documents (e.g., AMKA, insurance, etc.).	40	41.7
I didn't understand what they were telling me.	23	24
Other	11	11.5
**For the provision of health promotion and care services to Roma citizens, do you have access to**:		
Guidelines/directions from a public agency	41	46.6
Tools such as brochures or other materials that facilitate communication	22	25.0
Guidelines/directions from a scientific organization	20	22.7
Other	18	20.5
Guidelines/directions from another organization (e.g., NGO)	16	18.2

AMKA: social insurance number; NGO: non-govermental organization

The most significant barrier identified was the lack of health literacy and difficulty in understanding prevention and diagnostic tests, with an average score of 4.1 points (SD = 1.1 points). This was followed by insufficient information about services for protecting abused women, with a mean score of 4 points (SD = 1 point), and the lack of continuous care, negative attitudes towards healthcare professionals, and lack of cultural sensitivity in services, with a mean score of 3.9 points (SD = 1 point). We assigned the same score to the lack of awareness about primary healthcare. Significant barriers also included difficulty understanding written and spoken language and refusal to register with a family doctor, both with a mean score of 3.8 points (SD = 1.3 points).

Further down the list were insufficient information on procedures in cases of unwanted pregnancy and abortion, with a mean score of 3.7 points (SD = 1.2 points), and insufficient information on maternity and child protection, with a mean score of 3.6 points (SD = 1.2 points). Bureaucratic issues had a mean score of 3.5 points (SD = 1.2 points), as did the lack of transportation, with a mean score of 3.2 points (SD = 1.2 points). Additional barriers included the lack of understanding of the healthcare system, with a mean score of 3.1 points (SD = 1.2 points), and discrimination, which was rated similarly (SD = 1.1 points). Fear of the police had a mean score of 3 points (SD = 1.3 points), while the cost of care and depression/fear/anxiety/other personal reasons had a mean score of 2.8 points (SD = 1.3 points and 1.1 points, respectively).

Finally, the least significant barriers were evaluated as the lack of confidence in navigating the healthcare system and the lack of help with childcare, both with a mean score of 2.7 points (SD = 1.2 points and 1.3 points, respectively).

Supplementary Table S7. Problems Faced by Roma Women in Accessing Healthcare Services

Problem	Mean (SD)	Median (IQR)
Lack of health literacy, difficulty understanding prevention and diagnostic tests	4.1 (1.1)	4 (4 — 5)
Insufficient information about services for protecting abused women	4 (1)	4 (3 — 5)
Lack of continuous care, negative attitude towards healthcare professionals, lack of cultural sensitivity in services	3.9 (1)	4 (3 — 5)
Lack of awareness of primary healthcare	3.9 (1.1)	4 (3 — 5)

Difficulty understanding written and spoken language	3.8 (1.3)	4 (3 — 5)
Refusal to register with a family doctor	3.8 (1.3)	4 (3 — 5)
Insufficient information on procedures in case of unwanted pregnancy and abortion	3.7 (1.2)	4 (3 — 5)
Insufficient information on maternity and child protection	3.6 (1.2)	4 (3 — 5)
Bureaucratic issues, legal regulations	3.5 (1.2)	4 (3 — 4)
Lack of transportation	3.2 (1.2)	3 (2 — 4)
They don't understand the healthcare system and how to access it	3.1 (1.2)	3 (2 — 4)
Discrimination (e.g., registration)	3.1 (1.1)	3 (2 — 4)
Fear of reporting to the police	3 (1.3)	3 (2 — 4)
Cost of care	2.8 (1.3)	3 (1 — 4)
Depression/fear/anxiety/other personal reasons	2.8 (1.1)	3 (2 — 3)
Lack of confidence in navigating the healthcare system	2.7 (1.2)	3 (2 — 3)
Lack of help with childcare	2.7 (1.3)	3 (1 — 4)

SD: standard deviation; IQR: interquartile range

Supplementary Table S8. Problems Faced by Healthcare Professionals in Providing Health Services

Problem	N	%
Roma do not comply with the rules - they use Emergency	85	85.0

Departments instead of TOMY, Communication Centers, and Healthcare Centers		
Visits outside working hours and unscheduled appointments	82	82.0
Illiteracy	76	76.0
Urban-civic issues (ID, AMKA, birth certificates)	67	67.0
Lack of continuous care	53	53.0
Language	35	35.0
Lack of knowledge of cultural differences	29	29.0
Discrimination/negative attitude towards Roma	29	29.0
Lack of mediators, especially for emergency situations	29	29.0
Fear	28	28.0

AMKA: social insurance number; ID: identity; TOMY: local health center

Supplementary Table S9. Collaboration of Healthcare Professionals with Other Professionals

**Service/Professional**	**N**	**%**
**Services**		
Hospital	49	51.6
Police	35	36.8
Other municipal social services	29	30.5
Health Center	22	23.2
Community Center with Roma Branch	20	21.1
TOMY	19	20.0

Community Center	12	12.6
Mental Health Services/Mental Health Center	6	6.3
Private doctors	6	6.3
Other	5	5.3
**Professionals**		
Social Worker	49	50.5
Nurse/Health Visitor	41	42.3
Roma Mediator	25	25.8
Administrative Staff	18	18.6
Psychologist	9	9.3
Psychiatrist	6	6.2
Other	3	3.1

TOMY: local health center

Supplementary Table S10. Frequency of Collaboration of Healthcare Professionals with Other Professionals

Professional	N	%	% Most of the time - Always
Roma Mediator	Never	47	52.8
	Almost never	10	11.2
	Most of the time	28	31.5
	Always	4	4.5
Social Worker	Never	25	27.5
	Almost never	25	27.5
	Most of the time	33	36.3

	Always	8	8.8
Police	Never	29	33.7
	Almost never	27	31.4
	Most of the time	27	31.4
	Always	3	3.5
Psychologist	Never	43	53.8
	Almost never	25	31.3
	Most of the time	10	12.5
	Always	2	2.5
Psychiatrist	Never	48	62.3
	Almost never	20	26.0
	Most of the time	9	11.7
	Always	0	0.0

Supplementary Table S11. Participants' Agreement/Disagreement Rates on Opinions Related to Roma

Statement/Opinion	N	%
Roma women know enough about the organization of healthcare services	Agree	23.5
	Disagree	68.4
	Don't know	8.2
Almost all Roma women are Christians	Agree	28.6
	Disagree	25.5
	Don't know	45.9
Roma pregnant women have a 'theatrical' way of showing pain	Agree	71.7
	Disagree	16.2
	Don't know	12.1
	Agree	68.7

Many Roma women do not want to undergo medical tests	Disagree	19.2
	Don't know	12.1
The traditional marriage of the Roma is recognized by the Greek state as equivalent to a notarial deed	Agree	7.0
	Disagree	39.0
	Don't know	54.0

Supplementary Table S12. Reasons Why Roma Women Do Not Approach Healthcare Professionals

Reason	N	%
They don't know where to go for their problem	55	57.3
They are not sure they will receive proper care	28	29.2
Other	24	25.0
They fear the reactions of healthcare professionals	20	20.8
They don't trust healthcare professionals	11	11.5
They fear the reactions of other patients	10	10.4

Supplementary Table S13. Participants' Responses Regarding the First Case Study

Response	N	%
I am concerned about her child	53	53.0
I feel sorry for her	43	43.0
I am concerned about her	38	38.0
I consider informing a child protection organization about her situation	35	35.0

I respect and understand her choice to remain pregnant under these conditions	31	31.0
I feel the need to consult someone with more experience in providing care to refugees/migrants	30	30.0
I understand why it's difficult for her to keep her scheduled appointment	23	23.0
I feel annoyed that she didn't show up for the second time	23	23.0
I can't understand why she wants to bring a child into the world under these conditions	21	21.0
At least I'm glad I can help her	17	17.0
I feel desperate because I have no idea how to help her	7	7.0
Other	5	5.0

Supplementary Table S14. Participants' Responses Regarding the Second Case Study

Statement	N	%
Pregnant women without AMKA have the right to access healthcare	Correct	69.4
	Incorrect	16.3
	Don't know	14.3
Mediation services for Roma are free	Correct	37.5
	Incorrect	6.3
	Don't know	56.3
	Correct	59.8
	Incorrect	1.0

When the child is born, they will receive Greek citizenship	Don't know	39.2
Given that X is Roma and an unmarried mother, the birth certificate of her newborn will not include the father's details	Correct	69.4
	Incorrect	7.1
	Don't know	23.5

AMKA: social insurance number

Supplementary Table S15. Participants' Responses Regarding the Third Case Study

Response	N	%
You will contact Social Services that support Roma to assist X and her child and find them a new place to live	48	49.0
You inform her of all the possibilities for social and other support for herself and her child	44	45.4
You limit your care to the duties defined by your institution and provide her with a letter containing all relevant information to give to the healthcare professional who will continue to care for her during the postpartum period	37	37.8
You will contact Municipal Services that support Roma to assist X and her child and find them a new place to live	36	36.7
You discuss with her the appropriate living conditions during the postpartum	30	30.6

period and how to achieve the best possible outcome in her case		
You inform the multidisciplinary team monitoring her health, as she is in a high-risk group for mental and physical health problems during the postpartum period	18	18.4
You suspect that X has been a victim of violence and discuss it with her	14	14.3
You write a letter highlighting the reasons why X needs to obtain an AMKA	12	12.2
Since there is no husband or partner, you do not discuss contraception with X	4	4.1
You didn't think of it earlier, but now you wonder if X will smoke during the postpartum period and refer her to a smoking cessation clinic	3	3.1

AMKA: social insurance number

Supplementary Table S16. Participants' Responses Regarding the Fourth Case Study Involving a Roma Woman

**Response**	**N**	**%**
You will contact Social Services that support Roma to assist X and her child and find them a new place to live.	54	56.3
You will call the police to help you locate the woman.	42	43.8
You will contact Municipal Services that support Roma to	38	39.6

assist X and her child and find them a new place to live.		
You will persist for a few more days with the phone number you have, and if no one answers, you leave the case.	24	25.0
